# Intranasal Zolmitriptan-Loaded Bilosomes with Extended Nasal Mucociliary Transit Time for Direct Nose to Brain Delivery

**DOI:** 10.3390/pharmaceutics13111828

**Published:** 2021-11-01

**Authors:** Mai M. El Taweel, Mona H. Aboul-Einien, Mohammed A. Kassem, Nermeen A. Elkasabgy

**Affiliations:** Department of Pharmaceutics and Industrial Pharmacy, Faculty of Pharmacy, Cairo University, Kasr El-Aini Street, Cairo 11562, Egypt; mona.abouleinien@pharma.cu.edu.eg (M.H.A.-E.); mohamed.kassem@pharma.cu.edu.eg (M.A.K.); nermeen.ahmed.elkasabgy@pharma.cu.edu.eg (N.A.E.)

**Keywords:** zolmitriptan, intranasal, bilosomes, sodium deoxycholate, mucoadhesive gel, brain targeting

## Abstract

This study aimed at delivering intranasal zolmitriptan directly to the brain through preparation of bilosomes incorporated into a mucoadhesive in situ gel with extended nasal mucociliary transit time. Zolmitriptan-loaded bilosomes were constructed through a thin film hydration method applying Box–Behnken design. The independent variables were amount of sodium deoxycholate and the amount and molar ratio of cholesterol/Span^®^ 40 mixture. Bilosomes were assessed for their entrapment efficiency, particle size and in vitro release. The optimal bilosomes were loaded into mucoadhesive in situ gel consisting of poloxamer 407 and hydroxypropyl methylcellulose. The systemic and brain kinetics of Zolmitriptan were evaluated in rats by comparing intranasal administration of prepared gel to an IV solution. Statistical analysis suggested an optimized bilosomal formulation composition of sodium deoxycholate (5 mg) with an amount and molar ratio of cholesterol/Span^®^ 40 mixture of 255 mg and 1:7.7, respectively. The mucoadhesive in situ gel containing bilosomal formulation had a sol-gel temperature of 34.03 °C and an extended mucociliary transit time of 22.36 min. The gelling system possessed enhanced brain bioavailability compared to bilosomal dispersion (1176.98 vs. 835.77%, respectively) following intranasal administration. The gel revealed successful brain targeting with improved drug targeting efficiency and direct transport percentage indices. The intranasal delivery of mucoadhesive in situ gel containing zolmitriptan-loaded bilosomes offered direct nose-to-brain drug targeting with enhanced brain bioavailability.

## 1. Introduction

Migraine is the most abundant type of neurological disorder; coupled with atypical serotonergic activity [1,2], it is considered the second major cause of disability, especially among young people [3]. A migraine is a special kind of headache, consisting of a throbbing and severe headache in one side of the head [4]. Migraine can be associated with aura or not, with the latter being the most common type. Any of these symptoms can be associated with different etiologies [5]. Migraine with aura causes the patient to see strange colors and lights, which is sometimes scary and in some cases can also, be associated with ischemic stroke [6].

Zolmitriptan is a potent second generation triptan which acts as a selective serotonin receptor agonist [2]. It is used as pain terminator in migraine and cluster headache treatment and is given to patients with migraine attacks, with or without an aura. Although the oral administration of zolmitriptan is the most common route of administration, it is associated with poor bioavailability (≈40%) owing to severe hepatic first pass effect, slow onset of action [7,8] and systemic side effects such as nausea, dizziness, paraesthesia, neck pain and tightness [9]. Generally, the oral administration of anti-migraine drugs can be inconvenient, especially in patients with associated with nausea and vomiting.

For these reasons, attempts have been made to deliver zolmitriptan through other routes in order to bypass the hepatic metabolism and provide fast onset of action with enhanced bioavailability [7,8]. Among those routes, nose to brain drug delivery is of pronounced interest. Nasal delivery is an easy and non-invasive route for controlling migraine attacks, and is considered to be an attractive route of administration. There are six branches of arteries serving the nasal cavity which can enhance the systemic absorption of drugs; additionally, the presence of the olfactory region can provide a means of direct brain targeting [10]. All of these factors, as well as the increased surface area of the nasal cavity, can aid in enhanced drug absorption.

In 2003, the first zolmitriptan nasal spray was approved by FDA. Nevertheless, the approved aqueous solution provided only similar bioavailability to oral tablets, due to the first pass effect the drug is subjected to after systemic absorption [11]. Therefore, the fabrication of efficient nasal formulations capable of delivering the drug in sufficient amounts to the brain while avoiding rapid mucociliary clearance and poor membrane penetration is highly necessary.

Bilosomes (bile salts containing niosomes) are nanovesicular carriers composed of a non-ionic surfactant bilayer along with bile salts [12]. The literature includes several studies reporting the use of bilosomes in the efficient oral delivery of drugs and vaccines [13,14] thanks to their ability to resist gastrointestinal enzymes and consequent conferring of protection to the loaded active ingredient [14,15]. Additionally, the nature of the vesicular wall is considered a major point of difference between bilosomes and niosomes; the structure of niosomes lacks the additional edge activators of like bile salts. Edge activators act by lowering the surface tension of the vesicular bilayer, resulting in its destabilization and the formation of deformable vesicles [16] with enhanced tissue penetration [17].

Bilosomes have been applied for transdermal [18], topical [19] and ocular drug delivery [20]. However, to the authors’ knowledge, no published research has studied the influence of intranasal application of drug-loaded bilosomes on drug absorption and brain targeting. Although bilosomes have not yet been explored for intranasal drug delivery, their nanosize, coupled with the non-ionic surfactants and bile salts in their structure, provide promising scenarios for utilizing them in nose to brain drug delivery.

To gain the maximum benefit from the developed intranasal bilosomes, the nasal mucociliary transit time should be extended to allow for more residence of the applied dosage form inside the nasal cavity, improving drug absorption. This can be achieved by loading the developed bilosomes into a mucoadhesive in situ gelling system with high viscosity capable of resisting rapid mucociliary clearance [21,22].

Therefore, the objective of this study was to fabricate zolmitriptan-loaded bilosomes by applying 3^3^ Box–Behnken design and trying different formulation factors as well as loading the optimal bilosomal formulation into a mucoadhesive in situ gelling system with extended nasal mucociliary transit time. A temperature-induced in situ gelling system which transforms into gel form at body temperature was formulated using poloxamer 407. To boost the nasal residence time of the in situ gelling system, a mucoadhesive polymer (hydroxypropyl methyl cellulose) was added. The influence of formulation variables on entrapment efficiency, particle size and zeta potential, in addition to drug release, were evaluated. Furthermore, biological evaluation for the potential of brain drug delivery was assessed in rats.

## 2. Materials and Methods

### 2.1. Materials

Zolmitriptan was kindly supplied and certified by Global Nabi Pharmaceuticals (GNP), 6th of October, Giza, Egypt (Batch number: ZT 18040001). Brij^®^ 35 (polyoxyethylene (23) lauryl ether), Brij^®^ O10 (polyoxylethylene (10) oleyl ether), cholesterol, hydroxypropyl methylcellulose (HPMC) (viscosity 1500–4500 cp at 37 °C), poloxamer 407, sodium deoxycholate, Span^®^ 20 (sorbitan monolaurate), Span^®^ 40 (sorbitan monopalmitate), Span^®^ 60 (sorbitan monostearate), Span^®^ 80 (sorbitan monooleate), Tween^®^ 65 (polyoxyethylene sorbitan tristearate), Tween^®^ 80 (polyoxyethylene sorbitan monooleate) and dialysis tubing cellulose membrane (molecular weight cutoff 14,000 g/mol) were procured from Sigma-Aldrich, St. Louis, MO, USA. Methylene blue was obtained from El-Nasr Pharmaceutical Chemicals Company, Abu-Zaabal, Cairo, Egypt. Normal saline was purchased from Otsuka Pharmaceutical Co., Egypt. Acetonitrile and formic acid (both of HLPC grade) were supplied by Romil Limited, London, UK and E. Merk, D-6100 Darmstadt, Germany, respectively. Torsemide was provided and certified by DELTA Pharma, Cairo, Egypt. The water used was distilled de-ionized water. The other chemicals were of reagent grade and used as provided.

### 2.2. Optimization of Bilosomes

#### 2.2.1. Preparation of Zolmitriptan-Loaded Niosomes

Zolmitriptan-loaded niosomes were formed using the thin film hydration method [23], with minor modifications. In brief, 20 mg of drug were mixed with a 200 mg equimolar mixture of cholesterol and a non-ionic surfactant; all were dissolved in 10 mL chloroform/methanol mixture (3:7 *v*/*v*) in a round-bottomed flask. The organic solvent was evaporated at 60 °C under vacuum utilizing a rotary evaporator (Rotavapor, Heidolph VV 2000, Burladingen, Germany) adjusted at 90 rpm for 30 min to confirm complete elimination of the organic solvent. Following this, the formed thin dry film on the flask inner wall was hydrated using 10 mL distilled water. The hydration step was performed under normal pressure in the presence of glass beads for 1 h at 60 °C, utilizing the rotary evaporator which revolved at 90 rpm. Following this, the hydrated film dispersion was sonicated for 2 min using a bath sonicator (Crest ultrasonics corp., Trenton, NJ, USA) to ensure the formation of homogenous dispersion without any aggregates, then kept overnight at 6 °C. Eight non-ionic surfactants having different HLB values were utilized to prepare eight different niosomal formulations. The used surfactants were Span^®^ 20, Span^®^ 40, Span^®^ 60, Span^®^ 80, Tween^®^ 65, Tween^®^ 80, Brij^®^ 35 and Brij^®^ O10.

##### Evaluation of the Prepared Zolmitriptan-Loaded Niosomes

For each of the prepared niosomal formulations, drug entrapment efficiency (EE), particle size (PS) and polydispersity index (PDI) were evaluated.

##### Assessment of Entrapment Efficiency (EE)

To assess the EE% of the formed niosomal formulations, 1 mL of niosomal dispersion was mixed with 9 mL ethanol followed by sonication for 5 min using a bath sonicator. Actual drug content was measured spectrophotometrically (model UV-1601 PC; Shimadzu, Kyoto, Japan) by assaying its UV absorbance at a wavelength of 285 nm, after suitable dilution.

Additionally, the same volume (1 mL) of the niosomal formulation was ultra-centrifuged at 15,000 rpm, 4 °C for 90 min by means of a cooling centrifuge (Beckman, Fullerton, Canada). The supernatant was discarded and the residue was dissolved in 10 mL ethanol under sonication for 5 min in a bath sonicator to quantify the amount of entrapped drug [24]. Zolmitriptan content was assayed spectrophotometrically as before. EE% was determined as follows:

EE % = (Amount of entrapped drug/Actual drug content) × 100

##### Assessment of Particle Size (PS) and Polydispersity Index (PDI)

Furthermore, the niosomal preparations were assessed for their PS and PDI values. The examined formulations were suitably diluted with distilled water (1:10 *v*/*v*) until reaching faint opalescence, followed by analysis via ZetaSizer Nano ZS (Malvern Instruments, Worcestershire, UK). Moreover, particle size distribution was assessed via the determination of PDI. The used cuvettes were quartz, and the refractive index was adjusted at 1.33.

##### Statistical Analysis

One-way ANOVA followed by the least significance difference (LSD) test was applied to compare the EE% of the prepared zolmitriptan-loaded niosomal formulations using SPSS 17^®^ (SPSS Inc., Chicago, IL, USA). Zolmitriptan-loaded niosomes with the highest EE% were chosen as the optimal drug-loaded niosomal formulation. Data were gathered from two different batches for each preparation. Means and standard deviations were calculated from triplicate test results for every batch.

#### 2.2.2. Preparation of Zolmitriptan-Loaded Bilosomes

The same method utilized to prepare zolmitriptan-loaded niosomes was applied to prepare drug-loaded bilosomes, with bile salt (sodium deoxycholate) added to the chloroform/methanol solvent mixture. The resultant organic solution was treated as in the preparation of drug-loaded niosomes. Fifteen formulations were prepared using different weights of sodium deoxycholate and cholesterol/Span^®^ 40 mixture, as well as different molar ratios of cholesterol: Span^®^ 40 according to the constructed Box-Behnken statistical design.

##### Optimization of Zolmitriptan-Loaded Bilosomes

A three-factor, three-level Box-Behnken design (3^3^) was constructed in order to explore the effects of formulation factors on the main properties of the fabricated bilosomes and optimize their composition, for which Design-Expert^®^ software (Stat-Ease, Inc., Minneapolis, MN, USA) was employed. The independent variables were the amount of sodium deoxycholate (*X*_1_; 5, 10, 15 mg), the amount of cholesterol/Span^®^ 40 mixture (*X*_2_; 100, 200, 300 mg), and the molar ratio of cholesterol: Span^®^ 40 (*X*_3_; 1:1, 1:5, 1:9 *w*/*w*). Entrapment efficiency (*Y*_1_), particle size (*Y*_2_), polydispersity index (*Y*_3_), zeta potential (*Y*_4_), total % of zolmitriptan released after 0.5 h (Q0.5 h; *Y*_5_), and drug release efficiency (*Y*_6_) were chosen to be the dependent variables. Regarding the optimized formulation, responses *Y*_1_, *Y*_5_ and *Y*_6_ were preferred to be maximized, whereas other responses (*Y*_2_, *Y*_3_ and *Y*_4_) were desired to be minimized. According to the designed study, 15 formulations were prepared; 13 of them contained the midpoints of the investigated factors, while the center formulation was formulated three times. Two batches were prepared and results were collected in triplicates for each batch. The Box-Behnken design is presented in Table 1 and the detailed composition of the prepared drug-loaded bilosomal formulations is given in Table 2.

##### Characterization of the Prepared Zolmitriptan-Loaded Bilosomes—Assessment of Entrapment Efficiency, Particle Size (PS), Polydispersity Index (PDI) and Zeta Potential (ZP)

The same methods and steps used for assessing the drug-loaded niosomes were followed for the determination of the EE%, PS and PDI of the bilosomes. Additionally, the surface charge of the formulated bilosomes was evaluated by determining the ZP so as to evaluate the physical stability of the examined samples using ZetaSizer Nano ZS.

##### Characterization of the Prepared Zolmitriptan-Loaded Bilosomes—In Vitro Release Studies

For each investigated formulation, the dialysis bag technique was applied [25], where a certain volume equivalent to 4 mg entrapped drug was transferred in a dialysis bag which was pre-soaked 12 h in distilled water. The filled dialysis bag was properly secured from both ends and then dipped in 100 mL phosphate buffered saline (PBS, pH 7.4) in an amber colored glass bottle to assure the sink conditions. The bottles were then put in a thermostatically controlled shaking water bath (GFL, Gesellschatt laboratories, Berlin, Germany) operating at 100 shakes per min at 37 °C ± 0.5. Three mL samples of the release medium were withdrawn at specified time intervals (0.5, 1, 2, 3, 4, 6 and 8 h) and immediately substituted by an equivalent volume of fresh PBS. Samples were assayed spectrophotometrically for zolmitriptan content at λ_max_ 285 nm. This experiment was repeated for zolmitriptan aqueous dispersion (4 mg/2 mL) to check the appropriateness of the utilized dialysis cellulose membrane. For comparison purposes, the total percentage of drug released after 0.5 h (Q0.5 h), as well as the release efficiency (*RE*), were calculated for each of the investigated formulations. The *RE* parameter was the area under the release curve, expressed as the percentage of the rectangle area represented by 100% release at the same time. The following equation was used for calculation of *RE%* [26]:

$$RE\%=\int_{0}^{t}\;y.dt/y100\;t\times 100$$

where the integral represents the area under the release curve until time *t*, and *y*_100_ is the area of the rectangle representing 100% release at equivalent time. The best kinetic model was determined by fitting the obtained release data to the zero-order, Higuchi and Korsmeyer-Peppas models [27,28].

### 2.3. Mucoadhesive In Situ Gelling System

#### 2.3.1. Preparation of Mucoadhesive In Situ Gelling System Containing the Optimal Bilosomes

The mucoadhesive in situ gelling system was fabricated using the cold method [29]. Different gelling systems were prepared using different polymers at different concentrations (Table S1). A 3^3^ general factorial design using Design-Expert^®^ software was utilized to investigate the effect of formulation variables. The optimal gelling system was selected based on the desirability function (0.705). The optimal zolmitriptan-loaded bilosomes was dispersed in an aqueous solution of hydroxypropyl methyl cellulose (HPMC, the mucoadhesive polymer). A calculated amount of poloxamer 407 was then added slowly under magnetic stirring (WiseStir, Daihan Scientific Company, Daihan, Chhattisgarh, India) adjusted at 100 rpm at room temperature. The prepared dispersion was preserved in a refrigerator at 6 ± 2 °C for 24 h to equilibrate and form a clear solution. The used concentration of HPMC was 0.5% *w*/*v* and that of poloxamer 407 was 17% *w*/*v*. Batches were prepared in duplicates and the mean± standard deviations were calculated for three measurements for each batch.

#### 2.3.2. Characterization of the Prepared Mucoadhesive In Situ Gelling System

The optimal zolmitriptan-loaded bilosomal formulation in the prepared mucoadhesive in situ gelling system was re-characterized for its EE%, PS, PDI and ZP as was previously done for the free bilosomes prior to incorporation into the gel system. The prepared mucoadhesive in situ gelling system containing the optimal bilosomal formulation was characterized for drug release parameters (Q0.5 h and *RE%*), sol to gel transition temperature, nasal mucociliary transit time and rheological constants (consistency index and flow index).

##### Assessment of Release Parameters of Zolmitriptan from the Prepared Mucoadhesive In Situ Gelling System

The in vitro release testing was conducted using the dialysis bag diffusion method for the cold in situ gelling system loaded with optimal bilosomes, as previously described under the in vitro release testing of bilosomes and the percentage of total zolmitriptan released after 0.5 h and *RE%* was calculated.

##### Assessment of Sol to Gel Transition Temperature and Time

To determine the sol to gel transition temperature of the prepared gelling system, the tilting method was used [30]. A test tube containing 2 mL aliquot of the formulated clear solution of the cold mucoadhesive in situ gel was covered with Parafilm^®^ and affixed in a water bath. The temperature of the water bath was elevated gradually, starting from 20 °C, in increments of 0.5 °C every 10 min. The content was visually inspected for gelation by tilting it by 90°. The gelation temperature of the investigated formulation was its sol to gel transition temperature.

The sol to gel transition time was investigated using the test tube inversion method [31]. In brief, 1 mL of the mucoadhesive in situ gelling system (sol) was transferred into a 5 mL stoppered test tube which was placed a thermostatically controlled water bath kept at 37 °C. The transition time was determined by tilting the tube every 10 s till no flow was detected.

##### Assessment of Rheological Constants

The rheological characteristics of the investigated gelling system were assessed using a cone and plate viscometer (Brookfield viscometer; type DVT-2). The plate was connected to a water bath to keep its temperature at 35 ± 0.1 °C. A half-mL sample of the investigated cold system was transferred to the plate. The shear rate was elevated gradually from 0.5 to 100 min^−1^ and the viscosity reading was recorded [32]. The power law constitutive equation was then applied to the results [33]:

*Ƞ* = *my ^n^***^−^**^1^

where *ƞ* is the viscosity (cp), *y* is the shear rate (s^−1^), and *m* and *n* are constants representing consistency and flow indices, respectively.

##### Transmission Electron Microscopy (TEM)

Morphological examination of the optimal zolmitriptan-loaded bilosomal formulation as well as the prepared mucoadhesive in situ gelling system containing it was performed in order to assess aqueous dispersion. TEM (Lecia Image, Wetzlar, Germany) connected to camera model TKC 1380 JVC (Victor Company, Tokyo, Japan) at an accelerating voltage of 80 kV was used. A sample drop was placed on a carbon-coated copper grid surface and left for one minute; any excess was dried by a tip of filter paper.

##### Differential Scanning Calorimetry (DSC)

DSC was applied to both the optimal zolmitriptan-loaded bilosomes and the mucoadhesive in situ gelling system containing them after lyophilizing both.

Samples were initially frozen at −20 °C then lyophilized for 24 h (Lyophilizer; Novalyphe-NL 500; Savant Instruments Corp., Holbrook, NY, USA) with pressure adjusted at 7 × 10^−2^ mbar and condenser temperature maintained at −45 °C. The reconstitution time of the lyophilized samples was assessed by adding the same volume of distilled water prior to the lyophilization process to the dried samples. The time recorded till the formation of dispersion free from any aggregates was taken as the reconstitution time [34].

DSC analysis was also performed for zolmitriptan powder, a physical mixture of the individual components of the bilosomes, and the individual components of the gelling system. About 4 mg of each sample was scanned separately in aluminum pans from 30 to 300 °C at a heating rate of 10 °C/min under an inert nitrogen flow of 25 mL/min.

##### Determination of Nasal Mucociliary Transit Time

The nasal mucociliary transit time was assessed by an in vivo method described by Lale et al. [35], with slight adjustments. Nine rats (weighing 200–250 g) participated in this study. Animal use in this study was in accordance with the National Institutes of Health Guide for The Care and Use of Laboratory Animals (NIH, publication No.85-23, 1996). Rats were divided into three equal groups (*n* = 3). Animals were anesthetized by intramuscular injection of sodium thiopental (7 mg/kg) at the beginning of the experiment. A volume of 10 µL of the prepared in situ gelling system loaded with the optimal bilosomes and containing methylene blue dye (5 mg/mL) was introduced into the right nostril of the animals of Group 1 using a micropipette. Swabs from the nasopalatine canal and pharynx were done via oral cavity (using a moistened cotton-tipped applicator) each minute post application, for a total period of 60 min. Each swab was visually examined for the blue color of the dye. The time taken for the appearance of the blue dye was recorded as the mucociliary transit time [30]. The experiment was also performed with the optimal bilosomes pre-mixed with methylene blue (Group 2) as well as with methylene blue solution in saline, which was used as a control (Group 3).

### 2.4. In Vivo Animal Study

#### 2.4.1. Study Design and Dose Administration

This study was conducted to compare the pharmacokinetic parameters of zolmitriptan when using three different treatments. The investigated treatments were the optimal zolmitriptan-loaded bilosomes (Treatment A), the mucoadhesive in situ gelling system containing it (Treatment B), and the drug solution (Treatment C). A non-blind, three-treatment, one-period, randomized parallel design was used. Ninety male rats (weighing 200–250 g) participated in this study. The protocol of the study was approved by the institutional review board, Research Ethics Committee, Faculty of Pharmacy, Cairo University (PI 2326). The rats were housed nine per cage at ambient temperature with free access to food and water and a 12 h light/dark cycle. The animals were categorized into three equal groups (*n* = 30). Group 1 received intranasal (IN) Treatment A, Group 2 received IN Treatment B and Group 3 received intravenous (IV) Treatment C (used as a control). The dose of zolmitriptan in all treatments was 5 mg/kg [36]. The study lasted for 12 h and the animals were visually inspected throughout the study period for any behavioral disorder or sign of illness. At time intervals 0 (pre-dose), 0.25, 0.5, 1, 1.5, 2, 4, 6, 8 and 12 h following administration, three rats from each group were mercy sacrificed and plasma and brain samples were collected from them. Blood was collected from the trunk of the sacrificed animals into heparinized tubes and centrifuged (using Centurion Scientific LTD. Centrifuge, West Sussex, UK) at 4000 rpm for 15 min at 25 °C to separate plasma. In addition, the dissected brain was washed with normal saline, cleaned of all attaching tissue/fluid, and then homogenized with saline (three-fold its volume) for 1 min at 24,000 rpm using Pro scientific Homogenizer, (Oxford, UK). Plasma and brain samples were preserved at −20 °C pending analysis.

#### 2.4.2. Chromatographic Conditions

A sensitive, selective and validated LC-MS/MS method for quantitative determination of zolmitriptan in plasma and brain tissues was adopted [37,38]. Torsemide was utilized as an internal standard. The detector was a triple quadrupole MS/MS (Waters Corp., Milford, MA, USA) and positive ionization mode was used for zolmitriptan and torsemide monitoring. Empower™ 2 CDS Software solutions was used for data acquisition and data integration. The used column was a reverse-phase column (C_18_, 50 × 2.1 mm, Waters Corp., Milford, MA, USA). The mobile phase was a mixture of acetonitrile and 0.1% aqueous solution of formic acid 4:1 (*v*/*v*). Isocratic chromatographic separation was done at 40 °C and a flow rate of 0.2 mL/min.

#### 2.4.3. Sample Preparation

A certain volume of plasma or homogenized brain sample (0.5 mL) was mixed with a 50 µL solution of torsemide in acetonitrile (100 ng/mL). The mixture was vortexed for 30 s and then centrifuged at 3000× *g* for 15 min. The supernatant was withdrawn and filtered through a 0.45 µm Millipore^®^ filter. A centrifugal vacuum concentrator (Vacufuge 5301, Eppendorf, Hamburg, Germany) was utilized to dry the filtrate at 40 °C, which was then reconstituted with 100 µL mobile phase. A volume of 20 µL of the reconstituted sample was injected into the column using the autosampler.

#### 2.4.4. In Vivo Brain and Systemic-Kinetic Studies

The main pharmacokinetic parameters of zolmitriptan from the three treatments were determined in both plasma and homogenized brain tissues. For plasma samples, the mean plasma concentration of the drug was illustrated against time, and the peak plasma concentration (*C_max_*) and time taken to reach it (*t_max_*) were calculated. The area under the zolmitriptan plasma concentration–time curve (*AUC_0–12 h_*) represents the total amount of drug in plasma over 12 h. *C_max_, t_max_, AUC_0–12 h,_ AUC_0–__∞_*, mean residence time (*MRT*, calculated by the trapezoidal rule), as well as the time for half the initial dose to be eliminated (*t_1/2_*) and the drug elimination rate constant (*k*) were calculated by non-compartmental pharmacokinetic models using Kinetica^®^ software (version 5.0, 2017, Thermo Fisher Scientific, Waltham, MA, USA. The values of absolute bioavailability of the drug in plasma from the applied IN Treatments (A and B) were calculated relative to an intravenous aqueous solution of drug in normal saline.

For brain samples, the mean concentration of the drug in the homogenized brain was plotted versus time, and the main pharmacokinetic parameters of the drug in brain tissues (*C_max_, t_max_, AUC _0–12 h_, t_1/2_* and *k*) were determined as was done for the plasma samples. The values of drug bioavailability in the brain from the applied IN Treatments (A and B) compared to IV solution (Treatment C) were also calculated. In addition, two indices were calculated to express zolmitriptan targeting to the brain. These indices were drug targeting efficiency (DTE) [30] and nose to brain direct transport percentage (DTP) [39]. These indices were determined from the respective equations:

$$\mathrm{DTE}\left(\%\right)=\left(\frac{\frac{B_{IN}}{P_{IN}}}{\frac{B_{IV}}{P_{IV}}}\right)\times 100\;\;$$

$$\mathrm{DTP}\left(\%\right)=\left(\frac{B_{IN}-B_{x}}{B_{IN}}\right)\times 100$$

where *B* and *P* are *AUC_0–12_* in brain and plasma, respectively, IN and IV stand for intranasal and intravenous application, respectively, and *B_x_* is the brain AUC portion contributed by systemic circulation through the blood–brain barrier (BBB) subsequent IN application. *B_x_* was determined as follows [39]:

$$B_{x}=\left(\frac{B_{IV}\times P_{IN}}{P_{IV}}\right)\times 100$$

#### 2.4.5. Statistical Analysis

One-way ANOVA was carried out using SPSS 17.0 software followed by LSD test. Differences were significant at *p* < 0.05.

## 3. Results and Discussion

### 3.1. Optimization of Bilosomes

#### 3.1.1. Preparation of Zolmitriptan-Loaded Niosomes

As explained before, this study aimed at preparing zolmitriptan-loaded non-ionic surfactant nano-vesicles for brain targeting. Non-ionic surfactant nano-vesicles consist of niosomes and bilosomes; the former is considered a precursor for the latter. In other words, preparing good bilosomes requires starting with good niosomes. A very important variable affecting the quality of the prepared niosomes is the type of non-ionic surfactant used. In order to choose a suitable surfactant for the preparation of zolmitriptan-loaded niosomes, eight non-ionic surfactants with different HLB values were used under fixed conditions to inspect the effect of surfactant type on the EE of the formed vesicles. The investigated surfactants covered an HLB range of 4.3–16.0.

##### Evaluation of the Prepared Zolmitriptan-Loaded Niosomes

As entrapment of the water-soluble drug (zolmitriptan) in niosomes, which are lipid-rich vesicles, was the main challenge in the preparation of such vesicles, EE% was set as the evaluation criterion for the quality of the prepared niosomes.

Table 3 represents the EE% of zolmitriptan-loaded niosomes prepared using different surfactants. It can be seen that the EE% of vesicles prepared using any of the sorbitan esters was significantly higher than that prepared using any of the Tweens or Brijs as a surfactant. The EE% of niosomes prepared using sorbitan esters ranged from 14.90 ± 0.70% to 44.70 ± 1.17%, whereas the highest EE% of niosomes prepared using any of the Tweens or Brijs did not exceed 11.90 ± 1.65%. Differences in EE% between different surfactant types were found to be significant (*p* < 0.05). This could be ascribed to the different HLB values of the used surfactants and, consequently, the difference in their lipophilicity [40,41]. Values of EE% were found to increase as the HLB value of the used surfactant decreased (i.e., lipophilicity increased). Tweens and Brijs have HLB values > 10, indicating more hydrophilicity, which caused the vesicular membrane to be more permeable and leaky to the drug, leading to lower EE% values compared to vesicles prepared using sorbitan esters (with low HLB values ranging from 4.3–8.6).

Among the niosomes prepared using different sorbitan esters as surfactants, it was noticed that of EE% values of niosomes prepared using Span^®^ 40 or Span^®^ 60 (44.42 ± 1.17% and 33.33 ± 1.55%, respectively) were significantly higher (*p* < 0.05) than those prepared using Span^®^ 20 or Span^®^ 80 (14.90 ± 0.7% and 24.60 ± 1.35%, respectively). This could be related to the intrinsic properties of the sorbitan ester type used, especially the phase transition temperature. Span^®^ 40 and Span^®^ 60 are solid at ambient temperature due to their relatively high phase transition temperatures compared to Span^®^ 20 or Span^®^ 80. The stated phase transition temperatures are 48 and 53 °C versus 16 and −12 °C, respectively [40]. This temperature affects the nature of the surfactant bilayer being either in liquid or gel state, with the latter state occurring at higher phase transition temperatures. This gel state is characterized by the ordered arrangement of the surfactant alkyl chain, and hence leads to formation of less leaky bilayers [42].

Finally, by relating the EE% values of the formed niosomes utilizing either Span^®^ 40 or Span^®^ 60, it was evident that Span^®^ 40 excelled significantly (*p* < 0.05). This might be attributed to the relatively higher surface free energy of Span^®^ 40 (associated with higher HLB values) leading to the formation of vesicles of larger particle size with enhanced capability in terms of entrapping water-soluble drugs [43,44].

The relationship between PS and EE% was further confirmed by measuring the PS values of the investigated niosomes. The PS values of the prepared niosomes ranged from 308.31 ± 3.42 to 490.29 ± 6.53 nm, indicating the formation of nanovesicles. By comparing the PS of niosomes prepared with either Span^®^ 40 or Span^®^ 60, it was obvious that the former led to the formation of larger PS values compared to the latter, which could explain the enhanced EE% obtained with Span^®^ 40-based niosomes. The obtained PS values were 434.67 ± 9.75 and 383.56 ± 8.39 nm, respectively. The obtained PDI values of the prepared niosomes were between 0.213 ± 0.014 to 0.411 ± 0.007, indicating homogenous dispersion with low PDI values [45].

It can be concluded from the previous discussion that Span^®^ 40 was the surfactant that succeeded in enhancing the EE% of the drug. The EE% of niosomes prepared using Span^®^ 40 was quite satisfactory, as it is logical that entrapment of a water soluble drug in lipid niosomes is a great challenge. Accordingly, a niosomal formulation prepared using Span^®^ 40 was chosen to complete this study and prepare bile salt-enriched zolmitriptan-loaded niosomes (i.e., bilosomes) for further investigation.

#### 3.1.2. Investigation of the Effect Process Variables on the Properties of Zolmitriptan-Loaded Bilosomes

Statistical design was needed to correlate the main properties of the prepared drug-loaded bilosomes to the different formulation factors, to study the interaction between the investigated factors, and finally to suggest a formulation with optimized composition. A Box–Behnken (3^3^) design was constructed to optimize the composition of the zolmitriptan-loaded bilosomes. The pre-determination of the used surfactant during the preparation of the niosomes reduced the number of investigated formulation factors. In addition, the total number of 15 test runs needed for Box–Behnken design is fewer than that needed for a central composite design with the same number of repeats (17 runs) or that needed for a 3^3^ factorial design without repeats (27 runs) [46]. In addition, the used design enables studying of the interactions between different factors. For this reason, a Box–Behnken design was chosen in order to optimize the composition of the prepared bilosomes. The main output data of the applied design indicated good correlation between the predicted and adjusted *R*^2^ values for all responses with differences between them not exceeding 0.2, which confirmed that the applied design was appropriate for predicting response values [47]. Additionally, adequate precision for all the investigated responses exceeded the value of four; which is the desirable value. As adequate precision measures the signal to noise ratio, the results indicated that the applied model can be utilized for navigating the design space [48]. Furthermore, it can be concluded that the statistical model designed to be applied in this study is a valid one (Table S2).

##### Effect on Entrapment Efficiency (EE)

All the investigated bilosomes had a drug content approximately equal to 100%, as the actual drug content did not show any significant difference from theoretical one (20 mg). This indicated that there was no significant drug loss during preparation.

**Figure 1:**
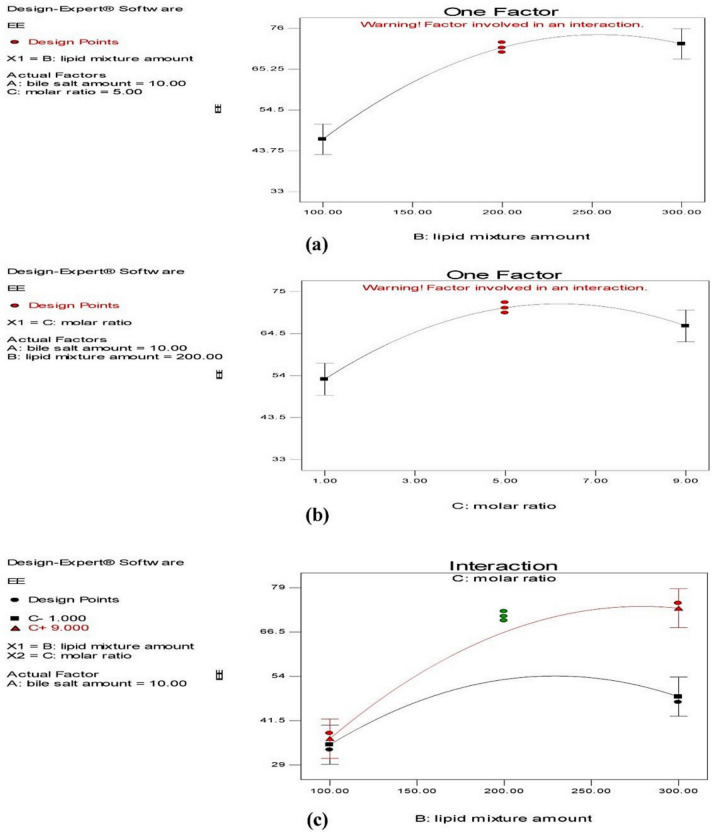
Line plots for the effects of cholesterol/Span^®^ 40 mixture amount (**a**) and cholesterol: Span^®^ 40 molar ratio (**b**), and the interactions between them (**c**) on entrapment efficiency of the prepared zolmitriptan-loaded bilosomes.

As revealed in Table 4, the EE% of the investigated zolmitriptan-loaded bilosomes ranged from 33.53 ± 1.59 to 74.33 ± 1.32% for formulations F9 and F12, respectively. ANOVA testing showed a non-significant effect of sodium deoxycholate amount (*X*_1_) on the EE% (*Y*_1_) of bilosomes (*p* = 0.0815) and significant effects of both the amount (*X*_2_) and molar ratio (*X*_3_) of cholesterol/Span^®^ 40 mixture (*p* < 0.0001 and = 0.0017, respectively), with a significant interaction between the two effective factors (*p* = 0.018). The individual effects of the two effective formulation factors on the EE% of the prepared bilosomes, as well as their interaction plot, are demonstrated in Figure 1. From Figure 1a, it is clear that increasing the amount of cholesterol/Span^®^ 40 mixture resulted in a significant increase in the EE% of the formed bilosomes. This finding might be attributed to the fact that the cholesterol/non-ionic surfactant mixture represents the backbone and the main vesicle-forming material of bilosomes; thus, increasing amounts might lead to the construction of a larger number of large-sized vesicles, and thus inclusion of larger drug amounts [49]. In addition, at low cholesterol/Span^®^ 40 mixture amounts, decreasing Span^®^ 40 level might lead to more drug leakage, as decreased Span^®^ 40 amounts are not adequate to stabilize the bilosomal membrane [50].

Figure 1b reveals that increasing the proportion of cholesterol in the cholesterol/Span^®^ 40 mixture (in other words, moving from cholesterol: Span^®^ 40 molar ratio 1:9 to 1:1) resulted in a significant reduction in their EE%. This is evident as the values of EE% of bilosomes of formulations F7, F8, F11 and F12 (containing a proportion of cholesterol = 10% of its mixture with Span^®^ 40) were significantly larger than those for the corresponding formulations with higher proportions of cholesterol (=50%), which were formulations F5, F6, F9 and F10. This might be attributed to the competition between cholesterol and the drug for the packing space in bilosomes prepared with relatively high proportions of cholesterol, which disrupted the regular bilayer structure and caused drug leakage [51,52]. In addition, the presence of large amount of cholesterol might increase the hydrophobicity of the interfacial region of the bilayers during the bilosomal preparation which disfavored the entrapment of the hydrophilic zolmitriptan [52]. On the other hand, in formulations with smaller proportions of cholesterol in the mixture with Span^®^ 40, the relatively high proportion of non-ionic surfactant increased the solubility of zolmitriptan in the organic phase during bilosomal preparation and decreased their escape to the aqueous phase, leading to enhanced drug entrapment.

The interaction between the two effective formulation factors (*X*_2_ and *X*_3_) on the EE% of the formed bilosomes is provided in Figure 1c. It is clear that at a cholesterol/Span^®^ 40 molar ratio 1:9, increasing the amount of the mixture resulted in increasing the EE%.

##### Effect on Particle Size (PS)

**Figure 2:**
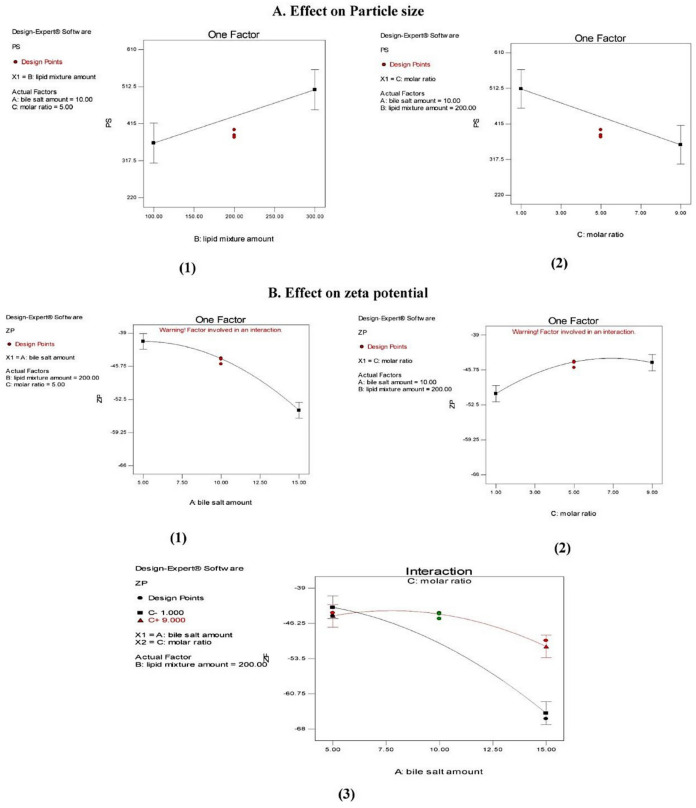
(**A**) Line plots of the effects of cholesterol/Span^®^ 40 mixture amount and (1) cholesterol: Span^®^ 40 molar ratio (2) on particle size. (**B**) Line plots for the effects of sodium deoxycholate amount (1), cholesterol: Span^®^ 40 molar ratio and (2) the interaction between them (3) on zeta potential of the prepared zolmitriptan-loaded bilosomes.

Table 4 demonstrates the mean PS values of the formed zolmitriptan-loaded bilosomes. The PS values were between 230.53 ± 12.62 nm (formulation F2) and 602.20 ± 13.21 nm (formulation F4). ANOVA testing indicated a non-significant effect (*p* > 0.05) of sodium deoxycholate amount (*X*_1_) on the mean PS as well as significant effects of both the amount of cholesterol/Span^®^ 40 mixture (*X*_2_) and the molar ratio (*X*_3_) between its components (*p* = 0.026 and 0.017, respectively); however, no interaction between the effective factors was indicated. The effects of the investigated formulation factors on PS are presented graphically in Figure 2.

From Figure 2(A1) it is clear that increasing the amount of cholesterol/Span^®^ 40 mixture from 100 mg (in formulations F1, F2, F9 and F11) to 300 mg in the corresponding formulations (F3, F4, F10 and F12) led to a significant increase in the PS of the prepared bilosomes. There are different explanations for this finding. As mentioned before, cholesterol/Span^®^ 40 mixture is the building unit of bilosomes; thus, increasing its amount enhanced the formation of larger number of large-sized vesicles [49,52,53]. Another explanation relates the larger size of the bilosomes prepared using larger amount of cholesterol/Span^®^ 40 mixture to their higher EE%. In addition, as water-soluble drugs like zolmitriptan favor entrapment in the vesicles’ aqueous core, this might cause a mutually repulsive interaction between the drug and the polar heads of the surfactant, leading to an increase in PS [43,54].

From Figure 2(A2) it can be seen that increasing the proportion of cholesterol in the cholesterol/Span^®^ 40 mixture resulted in a significant increase in the PS of the produced bilosomes. We believe that this is due to the packing of cholesterol molecules between the surfactant alkyl chain, leading to larger vesicular size [43,55].

##### Effect on Polydispersity Index (PDI)

PDI is a numerical value ranging from zero to one used to express the homogeneity of particle size distribution. When this index approaches zero, this indicates highly monodispersed particles, whereas, when it approaches one, this suggests highly polydispersed vesicles [56,57]. As presented in Table 4, the PDI of all the formed bilosomes ranged from 0.012 ± 0.00 to 0.49 ± 0.07 (for formulations F15 and F2, respectively) which indicated narrow size distribution of the prepared bilosomes. ANOVA testing indicated that the PDI of the prepared vesicles was not significantly (*p* > 0.05) influenced by the studied factors.

##### Effect on Zeta Potential (ZP)

The ZP of zolmitriptan-loaded bilosomes was between −65.1 ± 2.05 and −39.3 ± 0.81 mV (for formulations F6 and F3, respectively), confirming that the formed bilosomes had sufficient negative charge to maintain electric repulsion between them and inhibit their aggregation, and hence, better stability [58]. This negative charge in all formulations was mostly due to the use of the anionic bile salt sodium deoxycholate [59]. The results of ANOVA testing demonstrated a non-significant effect (*p* > 0.05) of the amount of cholesterol/Span^®^ 40 mixture (*X*_2_) and significant effects of both sodium deoxycholate amount (*X*_1_) and cholesterol:Span^®^ 40 molar ratio (*X*_3_) (*p* < 0.0001 and =0.0009, respectively), with a significant interaction between the latter two effects (*p* = 0.0017). The individual effects of factors *X*_1_ and *X*_3_ on ZP of the prepared bilosomes, in addition to the line plot representing the interaction between them, are illustrated in Figure 2B. From Figure 2(B1), it is clear that increasing the sodium deoxycholate amount from 5 mg (in formulations F1, F3, F5 and F7) to 15 mg in the corresponding formulations (F2, F4, F6 and F8) led to a significant increase in the absolute ZP value of the resultant bilosomes, which may be due to the anionic nature of this bile salt [60].

Figure 2(B2) reveals that increasing Span^®^ 40 proportion in the cholesterol/Span^®^ 40 mixture in the prepared bilosomes resulted in a significantly less negative ZP values. This is evident in the values of ZP for formulations prepared using a cholesterol:Span^®^ 40 molar ratio of 1:1 (F5, F6, F9 and F10) being significantly more negative than the corresponding ones prepared using a molar ratio of 1:9 (namely, F7, F8, F11 and F12). This may be due to the shielding effect of the excess non-ionic surfactant. As the used non-ionic surfactant is hydrophilic in nature, it might reside on the vesicular bilayer surface, leading to a less negative ZP [60,61]. From Figure 2(B3), it is clear that at a low cholesterol/Span^®^ 40 molar ratio, increasing the sodium deoxycholate amount resulted in increasing the negative ZP of the produced bilosomes. This is logical because, as explained before, this bile salt is the main cause of the negative potential of the prepared bilosomes.

##### Effect on In Vitro Release Studies

**Figure 3:**
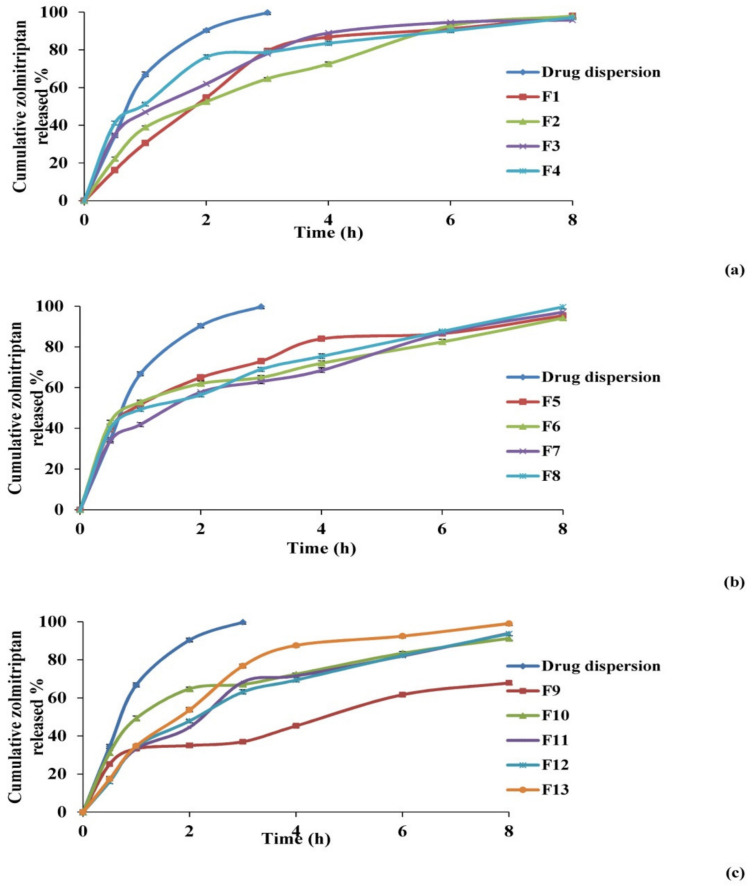
Release profiles of zolmitriptan from the prepared bilosomes compared to aqueous drug dispersion in phosphate buffer saline (pH 7.4); F1–F4 (**a**), F5–F8 (**b**) and F9–F13 (**c**).

The percentages of zolmitriptan released from the investigated bilosomes and drug aqueous dispersion are plotted versus time in Figure 3. The release profile of zolmitriptan from its dispersion revealed fast drug release, with about 99.8% of the drug released in the first 3 h. This assumed the free diffusion of drug through the dialysis membrane, indicating that the membrane used does not hinder drug availability in the release medium. Conversely, drug release from the prepared bilosomes was seen to be slower than that from its aqueous dispersion, which is quite logical.

Figure 3 also shows that the release of zolmitriptan from the investigated bilosomes was biphasic. An initial flush release of the drug took place within the first 0.5 h, followed by a slower release phase. The initial phase can be attributed to the fast release of adsorbed or surface drug in the release medium, owing to its hydrophilic nature [62], while the second phase of drug release was believed to be regulated by release through the swollen vesicle bilayers [63]. The biphasic release profiles of the drug from all the investigated bilosomes are expected to be of great benefit. Being an anti-migraine drug, the initial amount released would offer rapid onset of pain relief, while the slower subsequent release would keep the patient under prophylaxis from migraine over 8 h.

To compare zolmitriptan release from different drug-loaded bilosomes, two release parameters were applied: Q0.5 h and *RE%* from zero time to 8 h (Table 4). Q0.5 h percentage ranged from 15.5 ± 0.68% to 42.4 ± 0.75% for formulations F12 and F6, respectively. Furthermore, the lowest *RE%* result was recorded for F9 (46.5 ± 3.02%), while the highest was for F4 (77.3 ± 1.09%).

**Figure 4:**
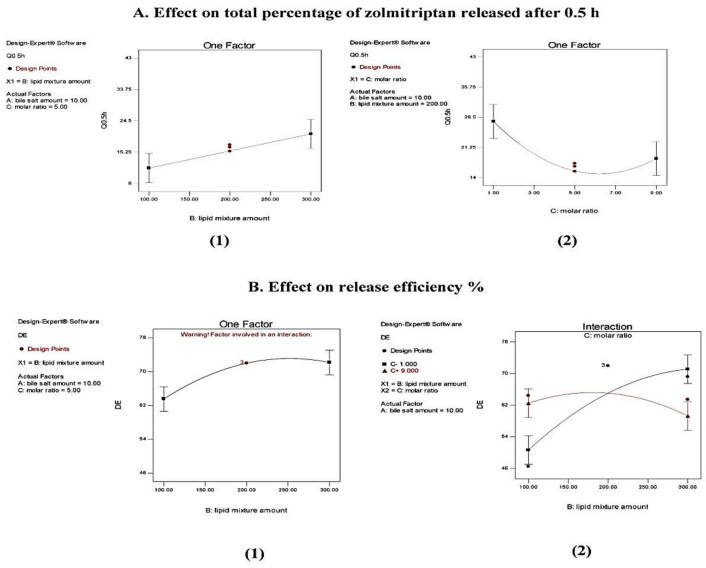
(**A**) Line plots of the effects of cholesterol/Span^®^ 40 mixture amount (1) and cholesterol: Span^®^ 40 molar ratio (2) on total percentage of zolmitriptan released after 0.5 h. (**B**) Line plots for the effect of the amount of cholesterol: Span^®^ 40 mixture (1) and the interaction between it and cholesterol: Span^®^ 40 molar ratio (2) on release efficiency % of the drug up to 8 h.

ANOVA analysis of Q0.5 h results showed a non-significant effect (*p* > 0.05) of sodium deoxycholate amount (*X*_1_) and significant effects of both cholesterol/Span^®^ 40 mixture amount (*X*_2_) and the molar ratio (*X*_3_) between components (*p* = 0.0109 and 0.0202, respectively), with no interaction between effective factors. The effect of the investigated formulation variables on Q0.5 h is represented graphically in Figure 4.

Regarding the effect of the amount of cholesterol/Span^®^ 40 mixture on Q0.5 h of the prepared bilosomes, it was evident that formulations containing larger amounts of cholesterol/Span^®^ 40 mixture (for example, formulations F3, F4, and F10) possessed higher Q0.5 h values than those for the corresponding formulations containing smaller amounts (F1, F2 and F9), as illustrated in Figure 4(A1). This can be credited to the significant elevation in EE% of the prepared bilosomes by increasing the amount of incorporated cholesterol/Span^®^ 40 mixture (as previously discussed), which was associated with increased driving force for the drug to be released, in line with Fick’s first law [64].

Figure 4(A2) shows that increasing cholesterol proportion from 10% to 50% in the cholesterol: Span^®^ 40 molar ratio 1:9 and 1:1 in formulations F7, F8, F11 and F12 compared to the corresponding formulations F5, F6, F9 and F10 led to a significant increase in the Q0.5 h values. This finding might be explained by the ability of cholesterol to disrupt the bilosomal membrane linear structure, as previously discussed [51,52]. This logically led to drug leakage, and hence enhanced drug release and higher Q0.5 h values.

It is obvious that using Q0.5 h as a release parameter involves comparison of single release points in order to evaluate the ability of the investigated formulations to release a fast initial dose of the loaded drug. On the other hand, the concept of *RE%* has some advantages over the single point parameter, the most important being that it can be theoretically related to in vivo data [65].

Concerning *RE%*, ANOVA results showed a non-significant effect (*p* > 0.05) of both the amount of sodium deoxycholate (*X*_1_) and cholesterol/Span^®^ 40 molar ratio (X_3_), and a significant effect of the amount of cholesterol/Span^®^ 40 mixture (*X*_2_) (*p* = 0.0021). The effect of the variable *X*_2_ is illustrated in Figure 4B. From Figure 4(B1) and Table 4, it is clear that increasing the amount of cholesterol/Span^®^ 40 mixture from 100 mg to 300 mg caused a statistically significant increase in *RE%*, as revealed by comparing the results obtained from formulations F1, F2 and F9 with those obtained from the corresponding formulations F3, F4 and F10. The increase can be attributed to the enhancement in terms of EE% of these bilosomes (as previously discussed), leading to the increase in drug concentration gradient and consequently to the enhancement of drug release. Statistical analysis also revealed a significant interaction between the amount of cholesterol/Span^®^ 40 mixture and the molar ratio between them (*p* = 0.0026). The line plot of the interaction presented in Figure 4(B2) shows that at a cholesterol: Span^®^ 40 molar ratio of 1:1, when the amount of this mixture was increased from 100 mg to 300 mg, the calculated value of *RE%* increased.

The drug release data presented best fit with the Korsmeyer-Peppas model, with correlation coefficient values (*r*) above 0.96.

#### 3.1.3. Selection of the Optimized Zolmitriptan-Loaded Bilosomes

The target of the optimization process is to specify the optimum levels of any variable required for the preparation of a pharmaceutical product with high quality. In this work, Design-Expert^®^ software was used to employ a numerical optimization approach, using the desirability function to overcome the multiple and opposing responses [66]. When applied, this numerical analysis developed suggested zolmitriptan-loaded bilosomes with an overall desirability of 0.758. This formulation was suggested to be prepared using 5 mg sodium deoxycholate (*X*_1_) and 255 mg of cholesterol/Span^®^ 40 mixture (*X*_2_) with a cholesterol:Span^®^ 40 molar ratio of 1:7.7 (*X*_3_). To evaluate the optimization capability of the model generated according to the Box–Behnken design, the suggested zolmitriptan-loaded bilosomes were prepared and characterized as was done for the previously prepared bilosomes. For each response, the observed value of the suggested bilosomes was collected with the expected one as well as the residual values which represented the difference between the expected and observed values of each response (Table 5). The residual values of all responses were small (not exceeding 2.1), signifying the reasonability of the optimization process. Thus, the suggested zolmitriptan-loaded bilosomes could be nominated as the optimal one, and it was used for further investigation.

### 3.2. Mucoadhesive In Situ Gelling System

#### 3.2.1. Characterization of the Prepared Mucoadhesive In Situ Gelling System

The loaded bilosomes in the prepared mucoadhesive in situ gelling system had an EE of 73.63 ± 1.12%, PS of 417.56 ± 16.52 nm, PDI of 0.32 ± 0.01 and ZP of −41.6 ± 0.66. These values were found to be non-significantly different from those previously obtained by free bilosomal dispersion, and *p* values were calculated to be 0.061, 0.059, 0.072 and 0.081, respectively.

#### 3.2.2. Assessment of Release Parameters of Zolmitriptan from the Prepared Mucoadhesive In Situ Gelling System

**Figure 5:**
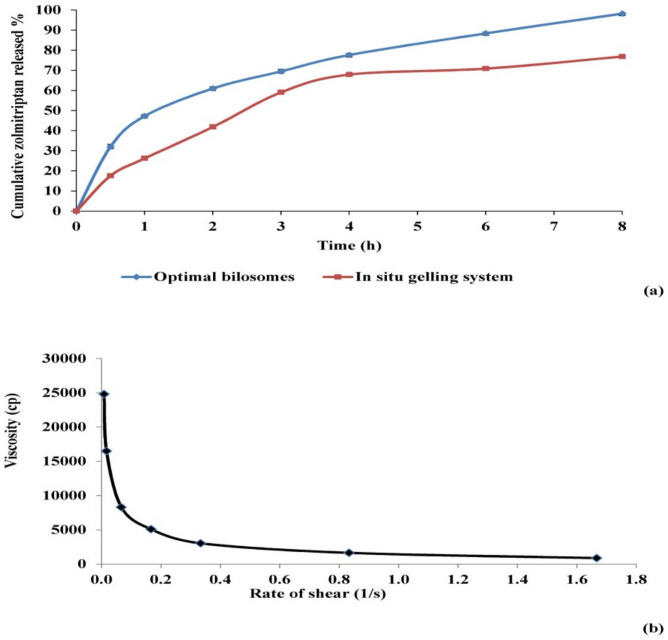
(**a**) Release profiles of zolmitriptan dispersion from the optimal bilosomes and the prepared mucoadhesive in situ gelling system containing them in phosphate buffer saline, pH 7.4; (**b**) Relation between viscosity and rate of shear for the prepared mucoadhesive in situ gelling system.

The drug release profiles of the prepared in situ gelling system as well as of the free optimal bilosomes are given in Figure 5a. The release best fitted the Korsmeyer–Peppas model, with an *r* value equal to 0.98. As shown in the figure, drug release from the prepared mucoadhesive in situ gelling system retained the biphasic profile given before from the prepared bilosomes, with an obvious reduction in the initial amount released. The Q0.5 h value of the drug released from the optimal bilosomes loaded in the gelling system decreased by around 50% compared to the free ones (*p* = 0.002). This might be due to the increased viscosity and density of the gelling system compared to free bilosomes, resulting in the slowing of drug diffusion and release [67,68,69]. Regarding the *RE%*, it was found that the difference between both the gelling system and free bilosomes was statistically non-significant (*p* > 0.05). It is worth to noting that in spite of the significant decrease in Q0.5 h of zolmitriptan from bilosomes when incorporated into the mucoadhesive in situ gelling system, the overall amount of drug released in a defined time (representing drug *RE*) did not display any significant change. In other words, the incorporation of drug-loaded bilosomes in the mucoadhesive in situ gelling system resulted in delayed drug release onset, with no effect on the total amount of drug released.

#### 3.2.3. Assessment of Sol to Gel Transition Temperature and Time

The sol to gel transition temperature of the prepared mucoadhesive in situ gelling system was 34.03 ± 0.45 °C. This transition temperature is close to the acceptable range for nasal administration, which has been stated by other researchers to be between 25 and 33 °C [30]. The sol to gel transition time was recorded to be 20.00 ± 1.00 s.

#### 3.2.4. Assessment of Rheological Constants

The rheological behavior of the mucoadhesive in situ gelling system loaded with optimal bilosomes was assessed using two indices, consistency and flow. Consistency index (*m*) represents the consistency or the thickness of the investigated fluid, which is more or less equivalent to the viscosity. On the other hand, flow index (*n*) represents the flow behavior of the fluid. When the value of *n* equals unity, this indicates Newtonian flow, while when it is bigger than one, the fluid shows dilatant flow (i.e., shear thickening). Values of *n* between zero and one indicate that the flow of the fluid is of a pseudo-plastic type, exhibiting shear thinning behavior [70,71,72].

For the prepared gelling system, the values of its viscosity at different shear rate values were reported at 35 °C, which represents the average temperature of nasal cavity. The calculated values of indices *m* and *n* were found to be 17717.40 and 0.39, respectively. The relatively large value of *m* indicated that the system is somewhat viscous, which could be explained by the plate temperature (35 °C) exceeding the sol to gel transition temperature of the investigated gelling system, leading to system gelation and an increase in its viscosity. As the value of *n* was less than unity, the investigated system exhibited shear-thinning flow. This flow is confirmed by the relation between viscosity and shear stress (Figure 5b). Such shear thinning behavior can be accredited to the decrease in viscosity by increasing shear rate [58,73]. The externally applied shear rate, responsible for the shear thinning behavior, could be achieved in vivo by the physiological ciliary movement, which is about 1000 beats per min [74]. This shear thinning behavior would allow for gradual release of the drug from the gel over time.

#### 3.2.5. Transmission Electron Microscopy (TEM)

Micrographs of all the examined samples showed that all samples were non-aggregating, almost spherical vesicles with well-defined walls and a smooth surface (Figure S1). This indicated that incorporation of the optimal zolmitriptan-loaded bilosomes in the mucoadhesive in situ gelling system used did not significantly affect their shape.

#### 3.2.6. Differential Scanning Calorimetry (DSC)

The lyophilized samples showed complete dispersion, with a reconstitution time of 16.67 ± 1.52 and 19.00 ± 1.00 s for the optimal bilosomal formulation and mucoadhesive in situ gelling system containing the bilosomal formulation, respectively. The thermal behavior of the pure drug, the lyophilized optimal bilosomes and the physical mixture of its components are demonstrated in Figure S2. A DSC thermogram of zolmitriptan exhibited a characteristic melting endothermic peak at 137.6 °C, signifying its crystalline nature. This peak could be observed in the physical mixture thermogram as well. However, this characteristic peak was not detected in the thermogram of the lyophilized optimal bilosomes, indicating the transformation of zolmitriptan to the amorphous form [75,76]. Additionally, the DSC thermograms of the prepared mucoadhesive in situ gelling system and the physical mixture of its components are presented in Figure S2. The characteristic drug peak can be noticed in the physical mixture thermogram but is not detected in the thermogram of the lyophilized mucoadhesive in situ gelling system, indicating the preservation of the drug-loaded bilosomal structure after being loaded inside the gelling system.

#### 3.2.7. Determination of Nasal Mucociliary Transit Time

Mucociliary clearance is the defense mechanism of the respiratory tract body against inhaled foreign substances. Hence, it is responsible for the quick clearance of nasally administered drugs to the nasopharynx, resulting in a significant decrease in their absorption [77]. In this study, nasal mucociliary transit time was assessed by measuring the time required by the applied system to reach the nasopalatine canal and the pharynx. The measured time expresses the time period during which the system can stay in the nasal cavity and thus available for drug absorption.

To avoid the difficulty of analyzing the drug in swabs taken from the nasopalatine canal and pharynx every minute, a blue dye (methylene blue) was mixed with the investigated samples to act as an indicator of the site it reached. In this study, the control solution (methylene blue in saline) appeared in swabs taken within the first minute of its application to the nasal cavity, indicating the successful administration of the dye solution to the laboratory animals.

The nasal mucociliary transit time of the optimal bilosomes was also determined and compared to results obtained from the prepared mucoadhesive in situ gelling system containing them in order to investigate the effect of incorporation in gelling system on their nasal mucociliary clearance. The nasal mucociliary transit time for dispersion of the optimal bilosomes was found to be 2.5 ± 0.71, versus 22.36 ± 0.41 min for the mucoadhesive in situ gelling system (*p* < 0.05). This is logical, as the prepared system is converted in the nasal cavity to a gel with higher viscosity and less clearance and, in addition, the incorporated mucoadhesive polymers increase the residence time of the prepared in situ gelling system in the nasal cavity by adhering to nasal mucosa [30].

### 3.3. In Vivo Animals

All the participating animals tolerated the drug, the examined preparations and the applied study design well, as they all remained alive and showed normal behavior and vitality until the final last sampling time. The applied LC/MS-MS method showed a lower limit of quantification (LLOQ) of 0.03 ng/mL in both plasma and brain tissues and showed good linearity, from 0.03 to 100 ng/mL (*R*^2^ of the corresponding lines were 0.9993 and 0.9990, respectively).

#### In Vivo Brain and Systemic–Kinetic Studies

**Figure 6:**
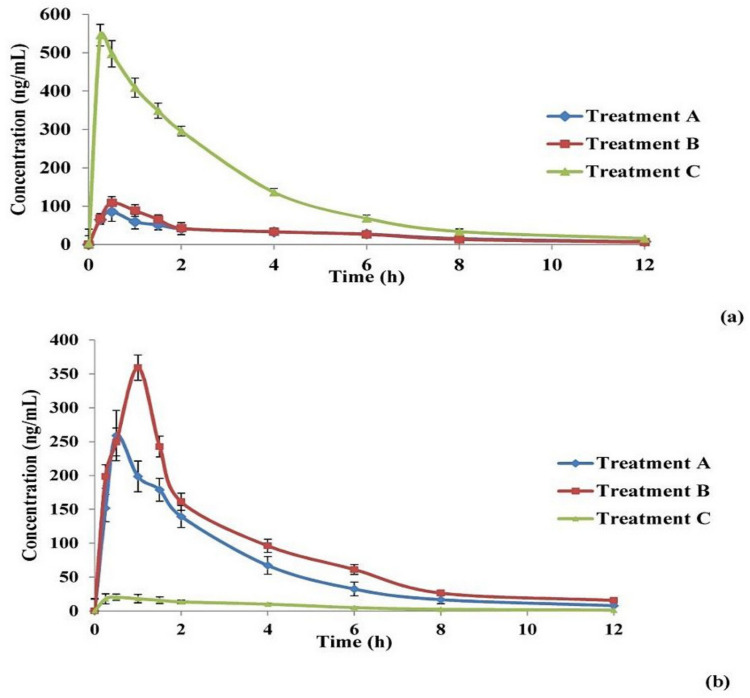
Zolmitriptan concentration versus time curves for plasma (**a**) and brain tissues (**b**) after administration of intranasal optimal bilosomes (Treatment A), intranasal mucoadhesive in situ gelling system (Treatment B), and intravenous zolmitriptan solution (Treatment C).

For each of the three applied treatments, zolmitriptan concentration in both plasma and homogenized brain was plotted versus time; the resulting curves are given in Figure 6.

It can be seen that the shapes of the zolmitriptan plasma concentration–time curves (Figure 6a) for bilosomes after IN administration either in the form of free bilosomes (Treatment A) or bilosomes contained in mucoadhesive in situ gel (Treatment B) obviously differed from that obtained for the IV drug solution (Treatment C). The former two treatments showed lower *C_max_* values and longer *t_max_* values. This indicates a difference in drug bioavailability between the IN treatments and the IV solution. The pharmacokinetic parameters of the drug from the three applied treatments are collected in Table 6. Zolmitriptan reached *C_max_* of 535.59 ± 9.04 ng/mL (after 0.25 h) when applied as an IV solution. The values of *C_max_* of the drug from the optimal bilosomes and the mucoadhesive in situ gel after IN administration were significantly lower (*p* < 0.05) than that for IV solution, being 108.58 ± 1.94 and 86.66 ± 4.13 ng/mL, respectively (both were reached after 0.5 h). Concerning the *AUC_0–12 h_* values under the plasma/time curve, it was found that the calculated values for Treatments A and B (361.86 ± 6.89 and 340.64 ± 36.48 ng·h/mL; respectively) were significantly smaller (*p* < 0.05) than that calculated for Treatment C (1579.32 ± 33.48 ng·h/mL). However, the difference in *AUC_0–12 h_* between Treatments A and B was statistically non-significant (*p* = 0.082). The same effect was obtained when *AUC_0-C_* was calculated, as revealed in Table 6. The obtained values with Treatments A and B might explain the availability of the drug in the brain tissues, which will be indicated by investigating brain kinetics. In addition, statistically non-significant differences (*p* > 0.05) were found between the three treatments concerning the time required to eliminate half the administered dose (*t_1/2_*) as well as in the elimination rate constant (*k*). The obtained values of drug *t_1/2_* and *k* ranged from 2.5 to 3 h and from 0.231± 0.006 to 0.277 ±0.003 h^−1^, respectively. These values were in agreement with those stated for zolmitriptan in the Electronic Medicines Compendium (EMC, Datapharm Ltd.) [78]. Regarding the MRT values, it was found that both Treatments A and B (4.08 and 4.80 h, respectively) possessed significantly larger values (*p* < 0.05) compared to Treatment C (3.33 h). Additionally, no significant difference in *MRT* values was detected between both Treatments A and B (*p* > 0.05). This could be due to the enhanced ability of the lipid nanovesicles like bilosomes to enhance nasal permeation of zolmitriptan through disrupting the mucosal membrane of the nose. Hence, due to the absorption process, the *MRT* of both Treatments A and B was found to be longer than that obtained after IV administration of the drug solution [11,79].

As revealed by the above plasma data, the values of absolute bioavailability of zolmitriptan in plasma from the optimal bilosomes (Treatment A) and from the mucoadhesive in situ gelling system (Treatment B) were 23.65 and 22.78%, respectively, with a statistically non-significant difference between them (*p* > 0.05). The small values of absolute bioavailability of the drug in plasma after administration of nasal bilosome treatments might be due to brain targeting of the drug. To confirm the brain targeting potential of the prepared bilosomes when applied IN, and to exclude any other factor that may result in poor plasma bioavailability of the drug, zolmitriptan concentration in homogenized brain tissues was estimated.

Zolmitriptan concentration in the homogenized brain samples was calculated and plotted versus time for the three treatments, as shown in Figure 6b. The curves of the three treatments are obviously different in shape. It is evident that Treatment B (IN mucoadhesive in situ gelling system containing the bilosomal formulation) showed the highest *C_max_*, followed by Treatment A (IN free bilosomal formulation), while Treatment C possessed the lowest *C_max_*. It is also obvious that the *t_max_* from Treatment B is the longest among the applied treatments. Table 6 shows the brain kinetic parameters of the applied treatments; the values of *C_max_* were 260.43 ± 6.90, 360.30 ± 7.78 and 21.13 ± 2.09 ng/mL for Treatments A, B and C, respectively, with significant differences between all of them (*p* < 0.05). Treatment B showed the longest *t_max_* (1 h) (*p* < 0.05) when compared to Treatments A and C (*t_max_* = 0.5 h). Table 6 also shows that the largest value of *AUC_0–12 h_* in brain was obtained by Treatment B (1081.88 ± 43.37 ng·h/mL), followed by Treatment A (768.24 ± 43.69 ng·h/mL), then Treatment C (91.92 ± 12.20 ng·h/mL), with significant differences (*p* < 0.05). The same effect between the three investigated treatments was obtained when comparing *AUC_0-C_* values. The t_1/2_ and k values of zolmitriptan from the three treatments showed non-significant differences (*p*-values < 0.05). By comparing the MRT values, it was found that the three treatments differed significantly from each other (*p* < 0.05), with Treatment B showing the highest MRT value (3.87 h) and Treatment C giving the least value (3.31 h). This extended MRT obtained with Treatment B compared to Treatment A might be attributed to the extended mucociliary transit time of the gelling system, which led to less clearance and hence better drug delivery to the brain through the olfactory duct [21].

As revealed from the brain data in Table 6, the brain bioavailability of the drug from IN optimal bilosomes (Treatment A) relative to IV solution (Treatment C) is 819.75% and that from IN mucoadhesive in situ gelling system (Treatment B) is 1173.64%. These results indicated that nasal application of the prepared bilosomes (either as dispersion or contained in mucoadhesive in situ gel) resulted in brain targeting of the incorporated zolmitriptan. The relative brain bioavailability of the drug from Treatment B was calculated to be 1.4-fold more than from Treatment A which might be accredited to the relatively long nasal mucociliary transit time of the mucoadhesive in situ gelling system (22.36 ± 0.41 min) as mentioned before. This long transit time enhanced the residence of the gelling system onto the nasal mucosa offering the time required for the drug to diffuse through the in situ formed viscous gel, hence better drug absorption [22,80]. This finding can explain the delayed *t_max_* of the drug in brain on administration of Treatment B.

In addition, the obtained values of *AUC_0–12 h_* (total drug amount) in both plasma and brain were compared using two parameters, drug targeting efficiency (DTE %) and direct transport percentage (DTP %), which were calculated. DTE expresses the result of dividing the ratio of total drug amount in brain to that in plasma for the IN treatment by the same ratio for the IV one. Thus, a 100% value of DTE represents an equal proportion of drug concentration in the brain for both the IN and IV routes of administration. If the value of the calculated DTE exceeds 100%, this represents preferential brain targeting of the drug from the IN route; if this value is less than 100%, it indicates preferential brain targeting from the IV route [30]. The DTE values for the applied IN Treatments (A and B) were calculated to be 3652.2 % and 5451.8% for Treatments A and B, respectively. Needless to say, the higher value of DTE calculated for Treatment B is due to the nature of its dosage form, as the prepared mucoadhesive in situ gelling system stayed at the site of application for longer time than bilosomal dispersion, leading to a higher *AUC_0–12 h_* and, consequently, a higher value of DTE. The calculated values of DTE (in addition to the obtained pharmacokinetic parameters) confirmed that the administered Treatments A and B resulted in favored zolmitriptan delivery to the brain compared to Treatment C. In other words, IN administration of the prepared zolmitriptan-loaded bilosomes resulted in boosted brain targeting with higher drug availability in the brain obtained by the prepared bilosome-containing mucoadhesive in situ gel.

To understand the possible pathways that the drug molecules go through to reach the brain, a brief overview of the anatomy of the nose should first be highlighted. The nasal cavity is divided into two main areas, respiratory and olfactory. The former is highly vascularized, while the latter is stimulated by the olfactory nerve [81].

Previous researchers have explained that there are two pathways for a drug to reach the brain when applied IN. The drug may be absorbed from the respiratory area of the nasal cavity into the general circulation through the nasal mucosa; it then permeates through the BBB to reach the brain. In the other pathway, the drug may be transported from the olfactory area of the nasal cavity to the olfactory bulb and then directly to the brain [82]. Although the olfactory epithelium constitutes only about 5% of the total area of the nasal cavity in humans [83], it is of significant interest when delivering drugs directly to the brain (bypassing the BBB) is required [84]. This unique feature of the olfactory epithelium is due to the existence of the olfactory neurons which are the receptors of the olfactory nerve. The olfactory nerve originates from the olfactory bulb, then reaches the olfactory area where olfactory neurons are embedded within its mucosa [85]. Additionally, there are two different mechanisms by which drug molecules can be transported from the olfactory area to olfactory bulb and then finally to different parts of the brain. These mechanisms are the intracellular and extracellular routes. Regarding the intracellular mechanism, the olfactory neurons internalize the drug molecule to be released later by exocytosis from neuron’s projection area. In the extracellular pathway, the drug initially crosses the epithelial membrane of the nose to the lamina propria area containing the neurons, located in the olfactory area of the nasal cavity [82].

To assess the relative contribution of the direct nose to brain pathway (i.e., olfactory pathway) in the overall brain delivery of zolmitriptan from the applied IN Treatments, the values of DTP % of the drug from Treatments A and B were calculated. A negative DTP value designates efficient drug delivery to the brain through the BBB, while a positive one designates a major contribution of the direct nose to brain pathway [30]. The calculated values of DTP were found to be 97.2 and 98.1% for Treatments A and B, respectively. The positive values of the calculated DTP indicated predominance of direct olfactory delivery of zolmitriptan to the brain for both bilosome treatments when applied in the nasal cavity. Enhanced DTE values as well as positive DTP values indicated the successful delivery of the drug to the brain tissues. These findings can be credited to the small particle size of the formed bilosomes, along with their elasticity. These properties promote both intracellular and extracellular drug transport to the brain, as previously mentioned. Their small size enabled the zolmitriptan-loaded bilosomes to be engulfed by the neurons of the olfactory nerve (i.e., intracellular drug transport), and the elasticity of the prepared bilosomes enabled them to squeeze and pass through the narrow extracellular route directly to the brain [86]. Enhancement of brain targeting of drugs when incorporated in nanocarriers for IN application has been reported by many researchers [11,87,88,89]. Moreover, it has been reported elsewhere that the presence of poloxamer 407 in the used in situ gel might lead to entanglement with the glycoprotein chains of the nasal mucosa, hence permitting better residence, absorption and penetration [90,91]. Additionally, the literature includes several examples showing the safety of the used components for intranasal applications, namely, cholesterol, Span^®^ 40 [92], sodium deoxycholate, and poloxamer 407 [93], and HPMC [94].

## 4. Conclusions

In the present study, zolmitriptan-loaded bilosomes were constructed using the thin film hydration technique, applying a Box–Behnken design after preliminary selection of the best non-ionic surfactant (Span^®^ 40). Sodium deoxycholate amount (5, 10 and 15 mg), cholesterol/Span^®^ 40 mixture amount (100, 200 and 300 mg) and the molar ratio between the mixture components (1:1, 1:5 and 1:9 *w*/*w*) were selected as independent factors. The optimum bilosomal formulation was suggested, after statistical analysis, to be made up of sodium deoxycholate (5 mg) and a cholesterol/Span^®^ 40 mixture of 255 mg at a molar ratio of 1:7.7 *w*/*w*. The selection was based on entrapment efficiency percentage, particle size, zeta potential and in vitro drug release. The optimum bilosomal formulation was loaded into a mucoadhesive in situ gel formed from a mixture of poloxamer 407 and HPMC. The mucoadhesive gel containing the bilosomal formulation possessed a sol–gel temperature of 34.03 °C and prolonged nasal mucociliary transit time of 22.36 min. Higher *C_max_* and *AUC_0–__∞_* coupled with longer t_max_ values in homogenized brain tissues revealed the superiority of the gel compared to free bilosomal dispersion (both administered intranasally), as well as the IV solution, in rats. Furthermore, the fabricated gelling system revealed successful brain targeting, which was confirmed by higher DTE and positive DTP values.

In conclusion, the developed formulation offered a promising intranasal substitute with boosted therapeutic effect for treating migraine suffering patients.

## Figures and Tables

**Table 1:** Design parameters and experimental conditions for Box–Behnken (3^3^) design.

Independent Variable (Factor)	Level		
	Low (−1)	Medium (0)	High (+1)
*X*_1_: Sodium deoxycholate amount (mg)	5	10	15
*X*_2_: Cholesterol/Span^®^ 40 amount (mg)	100	200	300
*X*_3_: Cholesterol: Span^®^ 40 molar ratio (*w*/*w*)	1:1	1:5	1:9
**Dependent variable (Response)**	**Desirability Constraint**		
*Y*_1_: Entrapment efficiency (%)	Maximize		
*Y*_2_: Particle size (nm)	Minimize		
*Y*_3_: Polydispersity index	Minimize		
*Y*_4_: Zeta potential (mV)	Minimize		
*Y*_5_: Q0.5 h (%)	Maximize		
*Y*_6_: Release efficiency (%)	Maximize		

Abbreviations; Q0.5 h (%), Total percentage of drug released after 0.5 h.

**Table 2:** Composition of the prepared zolmitriptan-loaded bilosomes.

Formulation Code	Composition		
	Sodium Deoxycholate (mg)	Cholesterol/Span^®^ 40 Mixture (mg)	Cholesterol: Span^®^ 40 Molar Ratio
	Midpoint Formulations		
**F1**	5	100	1:5
**F2**	15	100	1:5
**F3**	5	300	1:5
**F4**	15	300	1:5
**F5**	5	200	1:1
**F6**	15	200	1:1
**F7**	5	200	1:9
**F8**	15	200	1:9
**F9**	10	100	1:1
**F10**	10	300	1:1
**F11**	10	100	1:9
**F12**	10	300	1:9
**Center point formulations**			
**F13**	10	200	1:5
**F14**	10	200	1:5
**F15**	10	200	1:5

Drug concentration was kept constant (2 mg/mL) in all formulations.

**Table 3:** Entrapment efficiency percent of zolmitriptan-loaded niosomes prepared using different non-ionic surfactants with different HLB values.

Non-Ionic Surfactant		EE%
Name	HLB	
Span^®^ 20	8.6	14.90 ± 0.70
Span^®^ 40	6.7	44.72 ± 1.17
Span^®^ 60	4.7	33.33 ± 1.55
Span^®^ 80	4.3	24.60 ± 1.35
Tween^®^ 65	10.5	11.90 ± 1.65
Tween^®^ 80	15.0	6.50 ± 0.70
Brij^®^ 35	16.0	9.70 ± 0.70
Brij^®^ O10	12.0	8.80 ± 0.83

Results are expressed as mean values ± SD, Abbreviations; EE, Entrapment efficiency.

**Table 4:** Measured responses of Box–Behnken (3^3^) design for the prepared zolmitriptan-loaded bilosomes.

Formulation Code	Responses					
	EE (%)	PS (nm)	PDI	ZP (mV)	Q0.5 h (%)	RE (%)
F1	42.73 ± 1.19	343.33 ± 18.53	0.34 ± 0.03	−41.4 ± 1.15	16.0 ± 0.64	72.0 ± 3.09
F2	37.67 ± 1.33	230.53 ± 12.62	0.49 ± 0.07	−55.0 ± 1.27	22.3 ± 1.00	68.6 ± 2.06
F3	63.40 ± 0.74	448.72 ± 19.32	0.33 ± 0.04	−39.3 ± 0.81	34.8 ± 1.60	76.1 ± 3.04
F4	67.46 ± 0.77	602.20 ± 13.21	0.35 ± 0.04	−54.2 ± 1.50	39.6 ± 1.41	77.3 ± 1.09
F5	51.63 ± 1.22	560.50 ± 20.55	0.26 ± 0.01	−45.0 ± 1.48	38.5 ± 1.24	74.1 ± 1.07
F6	44.97 ± 1.35	527.00 ± 55.27	0.35 ± 0.04	−65.1 ± 2.05	42.4 ± 0.75	70.0 ± 3.08
F7	65.73 ± 1.66	409.27 ± 34.68	0.35 ± 0.04	−44.5 ± 0.50	32.8 ± 1.00	67.8 ± 1.06
F8	51.03 ± 1.59	460.43 ± 48.91	0.28 ± 0.02	−48.8 ± 0.95	38.4 ± 1.22	70.8 ± 2.05
F9	33.53 ± 1.59	531.23 ± 31.97	0.16 ± 0.03	−45.3 ± 0.60	24.5 ± 0.96	46.5 ± 3.02
F10	46.06 ± 1.45	597.96 ± 34.41	0.35 ± 0.04	−51.2 ± 0.70	31.3 ± 0.55	69.2 ± 1.07
F11	37.55 ± 0.83	356.97 ± 42.54	0.16 ± 0.03	−44.8 ± 1.05	18.1 ± 1.00	64.4 ± 3.03
F12	74.33 ± 1.32	352.23 ± 43.37	0.29 ± 0.03	−48.0 ± 0.50	15.5 ± 0.68	63.5 ± 0.14
F13	70.97 ± 1.30	384.40 ± 10.05	0.017 ± 0.00	−45.3 ± 0.64	15.5 ± 0.96	71.9 ± 0.19
F14	71.39 ± 1.30	379.30 ± 10.05	0.019 ± 0.00	−44.3 ± 0.64	16.7 ± 0.96	72.1 ± 0.19
F15	70.83 ± 1.30	398.20 ± 10.05	0.012 ± 0.00	−44.1 ± 0.64	17.4 ± 0.96	71.8 ± 0.19

Abbreviations; EE, Entrapment efficiency; PS, Particle size; PDI, Polydispersity index; ZP, Zeta potential; Q0.5 h, Total percentage of zolmitriptan released after 0.5 h; RE, Release efficiency.

**Table 5:** Optimal levels of factors of the suggested zolmitriptan-loaded bilosomes with the expected, observed and residual values of each response.

Factor		Optimal Level	
*X*_1_: Sodium deoxycholate amount (mg)		5	
*X*_2_: Cholesterol/Span^®^ 40 amount (mg)		255	
*X*_3_: Cholesterol: Span^®^ 40 molar ratio (*w*/*w*)		1:7.7	
**Response**	**Expected value**	**Observed value**	**Residual value ^a^**
*Y*_1_: Entrapment efficiency (%)	71.70	70.34 ± 0.10	1.36
*Y*_2_: Particle size (nm)	399.27	399.80 ± 4.95	−0.53
*Y*_3_: Polydispersity index	0.33	0.33 ± 0.05	−0.004
*Y*_4_: Zeta potential (mv)	−42.50	−41.90 ±0.19	−0.60
*Y*_5_: Q0.5 h (%) ^a^	31.87	32.20 ± 1.09	−0.33
*Y*_6_: Release Efficiency (%)	73.86	71.76 ± 0.34	2.10

^a^ Residual value = expected value—observed value.

**Table 6:** Pharmacokinetic parameters of zolmitriptan in plasma and brain after administration of intranasal optimal bilosomes (Treatment A), intranasal mucoadhesive in situ gelling system (Treatment B) and intravenous solution (Treatment C).

**In Plasma**			
**Parameter**	**Treatment A**	**Treatment B**	**Treatment C**
***C_max_*** **(ng/mL) ^a^**	108.58 ± 1.94	86.66 ± 4.13	535.59 ± 9.04
***t_max_*** **(h) ^b^**	0.5	0.5	0.25
***AUC_0–12 h_*** **(ng·h/mL) ^c^**	361.86 ± 6.89	340.64 ± 36.48	1579.32 ± 33.48
***AUC_0–∞_*** **(ng·h/mL) ^d^**	388.62 ± 4.77	370.43 ± 31.26	1643.89 ± 35.43
***K*** **(h^−1^)**	0.251 ± 0.010	0.231 ± 0.006	0.277 ± 0.003
***t_1/2_*** **(h)** ***MRT*** **^e^**	2.76 ± 0.15 4.08	3.00 ± 0.24 4.80	2.5 ± 0.38 3.33
Absolute bioavailability (%) *	23.65	22.78	---
**In Brain**			
**Parameter**	**Treatment A**	**Treatment B**	**Treatment C**
***C_max_*** **(ng/mL) ^f^**	260.43 ± 6.90	360.30 ± 7.78	21.13 ± 2.09
***t_max_*** **(h) ^g^**	0.5	1	0.5
***AUC_0–12 h_*** **(ng·h/mL) ^h^**	768.24 ± 43.69	1081.88 ± 43.37	91.92 ± 12.20
***AUC_0–∞_*** **(ng·h/mL) ^i^**	801.15 ± 46.94	1147.08 ± 51.79	97.73 ± 13.86
***K*** **(h^−1^)**	0.275 ± 0.011	0.25 ± 0.003	0.256 ± 0.003
***t_1/2_*** **(h)**	2.52 ± 0.18	2.77 ± 0.16	2.70 ± 0.31
***MRT*** **(h) ^j^**	3.36	3.87	3.31
Brain bioavailability (%) **	819.75	1173.64	----

Results are mean values ± SD, in plasma data and compared to Treatment C; ^a^ *p* < 0.05 (0.0015 and 0.0012 for Treatments A and B, respectively), ^b^ *p* < 0.05 (0.0072 for both Treatments A and B), ^c^ *p* <0.05 (0.0014 and 0.0011 for Treatments A and B, respectively). No significant difference was detected between Treatments A and B, ^d^ *p* < 0.05 (0.00138 and 0.00114 for Treatments A and B, respectively). No significant difference was detected between Treatments A and B, ^e^ *p* <0.05 (0.0017 and 0.00109 for Treatments A and B, respectively). No significant difference was detected between Treatments A and B. * *p* < 0. 05 (0.278 between Treatments A and B), In brain data and compared to Treatment C; ^f^ *p* < 0.05 (0.0021 and 0.0017 for Treatments A and B, respectively), ^g^ *p* < 0.05 (0.0074 for Treatment B), ^h^ *p* <0.05 (0.0023 and 0.0013 for Treatments A and B, respectively). No significant difference was detected between Treatments A and B, ^i^ *p*< 0.05 (0.0022 and 0.0014 for Treatments A and B, respectively). No significant difference was detected between Treatments A and B, ^j^ *p* < 0.05 (0.0023 and 0.0014 for Treatments A and B, respectively). No significant difference was detected between Treatments A and B. ** *p* < 0.05 (0.004 between Treatments A and B).

## Data Availability

The data presented in this study are available in the article and Supplementary Material.

## Supplementary Materials

The following are available online at https://www.mdpi.com/article/10.3390/pharmaceutics13111828/s1, Table S1: Composition and characterization of the mucoadhesive in situ gelling formulations prepared according to 3^3^ general factorial design using Design-Expert^®^ software. Table S2: Main output data of the Box–Behnken (3^3^) design for the analysis of zolmitriptan-loaded bilosomes F1–F15^a^. Figure S1: Transmission electron micrographs of the optimal zolmitriptan-loaded bilosomes (a) and the mucoadhesive in situ gelling system containing them (b). Figure S2: Differential scanning calorimetry thermograms of zolmitriptan (a), physical mixture of components of optimal zolmitriptan-loaded bilosomes (b), lyophilized powder of the optimal bilosomes (c), physical mixture of components of the prepared mucoadhesive in situ gelling system loaded with optimal bilosomes (d) and lyophilized mucoadhesive in situ gelling system loaded with optimal bilosomes (e). Supplementary Material S1: Effect of storage.
