# Supplementary material for: Intranasal Zolmitriptan-Loaded Bilosomes with Extended Nasal Mucociliary Transit Time for Direct Nose to Brain Delivery
Source: Pharmaceutics. 2021 Nov 1;13(11):1828. doi: 10.3390/pharmaceutics13111828 (PMC8624645; doi:10.3390/pharmaceutics13111828)
Supplement: Supplementary file 1 [file pharmaceutics-13-01828-s001.zip › pharmaceutics-1371914-supplementary.pdf]

pharmaceutics

MDPI

Supplementary Materials: Intranasal Zolmitriptan-Loaded Bilosomes with Extended Nasal Mucociliary Transit Time for Direct Nose to Brain Delivery

Mai M. El Taweel, Mona H. Aboul-Einien, Mohammed A. Kassem, Nermeen A. Elkasabgy

Table S1. Composition and characterization of the mucoadhesive in-situ gelling formulations prepared according to 3^3 general factorial design using Design-Expert® software.

Formulation Code	Composition			Responses					
	X_1: Poloxamer 407 Concentration	X_2: Type of Mucoadhesive Polymer	X_3: Concentration of Mucoadhesive Polymer	Y_1: Q0.5 h [b] (%)	Y_2: Sol-Gel T [c] (°C)	Y_3: RE [d] (%)	Y_4: Mucociliary Transit Time (min)	Y_5: Consistency Index (*m*)	Y_6: Flow Index (*n*)
G1	15%	HPMC	0.1%	16.65 ± 0.56	31.83 ± 0.30	67.45 ± 0.33	6.31 ± 0.23	11711.2	0.262
G2			0.3%	13.80 ± 0.30	28.40 ± 0.45	67.62 ± 0.26	7.68 ± 0.39	12240.0	0.540
G3			0.5%	12.08 ± 0.70	35.50 ± 0.45	47.94 ± 0.15	21.76 ± 0.47	13920.0	0.259
G4	17%		0.1%	18.10 ± 0.62	28.46 ± 0.45	57.67 ± 0.21	10.36 ± 0.23	13384.0	0.372
G5			0.3%	12.96 ± 0.67	25.63 ± 0.23	49.36 ± 0.24	11.63 ± 0.44	12159.0	0.440
G6 [a]			0.5%	17.58 ± 0.54	34.03 ± 0.45	56.36 ± 0.23	22.36 ± 0.41	17717.4	0.390
G7	15%	Na alginate	0.1%	23.8 ± 0.36	40.33 ± 0.41	69.41 ± 0.28	4.44 ± 0.09	131.2	1.014
G8			0.3%	9.89 ± 0.26	38.46 ± 0.45	49.48 ± 0.11	5.44 ± 0.13	214.2	1.014
G9			0.5%	10.76 ± 0.61	42.30 ± 0.28	59.71 ± 0.16	7.33 ± 0.11	165.5	1.012
G10	17%		0.1%	13.86 ± 0.61	37.16 ± 0.62	48.38 ± 0.31	6.49 ± 0.54	9377.7	0.160
G11			0.3%	6.50 ± 0.60	33.20 ± 0.28	49.74 ± 0.17	6.65 ± 0.41	1584.2	0.610
G12			0.5%	11.77 ± 0.72	40.16 ± 0.28	53.20 ± 0.34	8.38 ± 0.16	271.2	1.001

[a] G6 was selected as optimum gelling system according to desirability criteria in design expert software (= 0.705), [b] Percentage of zolmitriptan released after 0.5 h, [c] Sol-gel transition temperature, [d] Drug release efficiency.

Table S2. Main output data of the Box-Behnken (3^3) design for the analysis of zolmitriptan-loaded bilosomes F1- F15 [a].

Response [b]	Y_1	Y_2	Y_3	Y_4	Y_5	Y_6
Minimum	33.30	230.53	0.012	−65.1	15.5	46.5
Maximum	74.60	602.20	0.49	−39.3	42.4	77.3
Ratio	2.24	2.61	40.83	6.48	2.73	1.66
Model	Quadratic	Linear	Quadratic	Quadratic	Quadratic	Quadratic
R-squared	0.9653	0.5454	0.7188	0.9561	0.8492	0.9007
Adjusted R-squared	0.9306	0.4621	0.6719	0.9318	0.7888	0.8455
Predicted R-squared	0.8270	0.4758	0.6671	0.8244	0.7607	0.8809
Adequate Precision	13.850	8.367	9.206	21.701	10.326	14.969
Significant factors [c]	X_2, X_3	X_2, X_3	-	X_1, X_3	X_2, X_3	X_2

[a] Composition of the formulae is given in Table 2, [b] Nomination of responses (Y_1 – Y_6) is given in Table 1, [c] Nomination of factors (X_1–X_3) is given in Table 1.

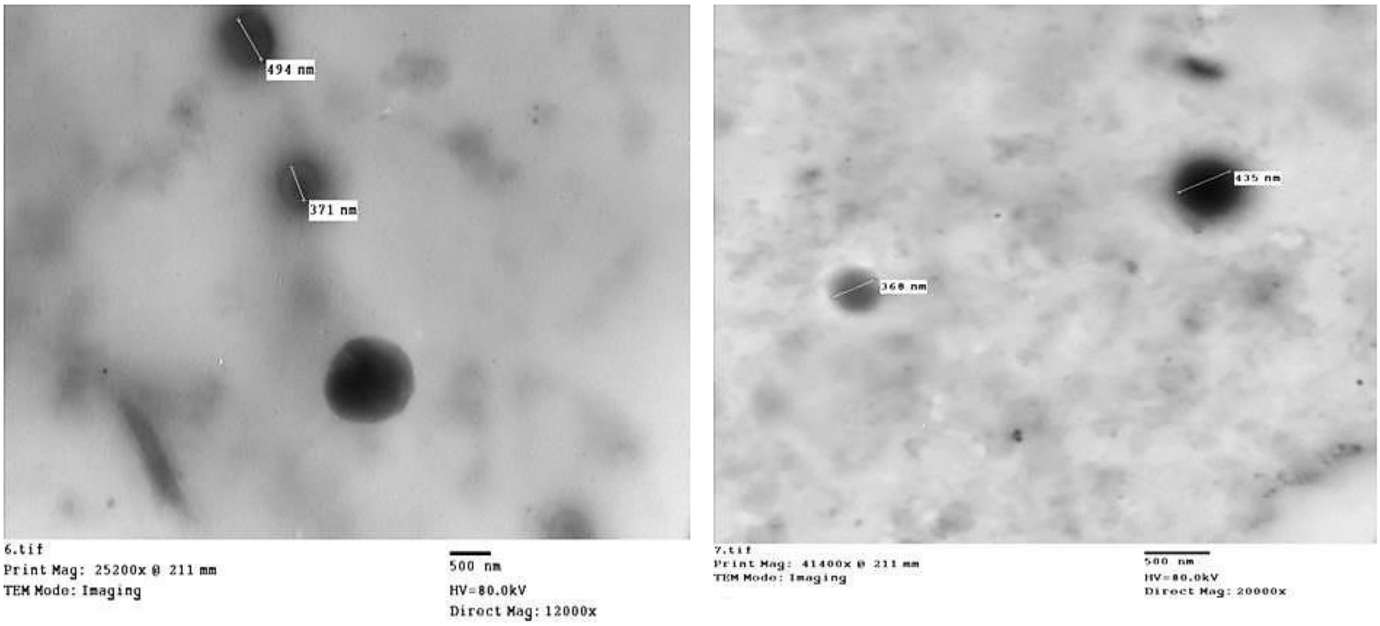

(a) (b)

Figure S1. Transmission electron micrographs of the optimal zolmitriptan-loaded bilosomes (**a**) and the mucoadhesive in-situ gelling system containing them (**b**) showing their particle size values.

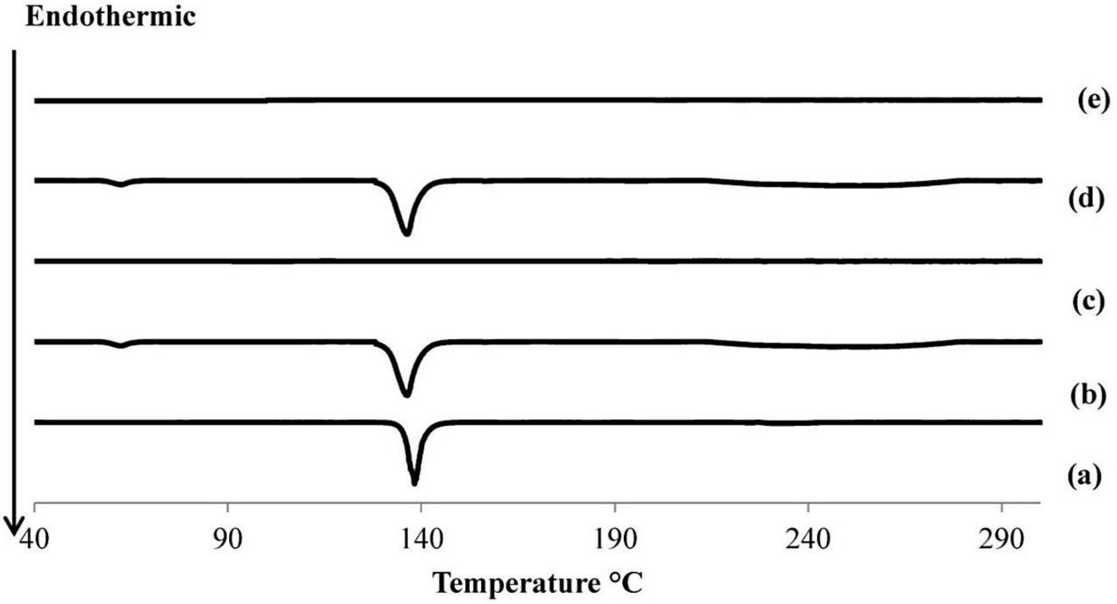

Figure S2. Differential scanning calorimetry thermograms of zolmitriptan (**a**), physical mixture of components of optimal zolmitriptan-loaded bilosomes (**b**), lyophilized powder of the optimal bilosomes (**c**), physical mixture of components of the prepared mucoadhesive *in-situ* gelling system loaded with optimal bilosomes (**d**) and lyophilized mucoadhesive *in-situ* gelling system loaded with optimal bilosomes (**e**).

Supplementary Material S1. Effect of Storage

Methods

The effect of storage on both the optimal zolmitriptan-loaded bilosomal dispersion and the prepared mucoadhesive *in-situ* gelling system containing it (sol) was investigated. Samples were separately transferred to sealed glass vials and stored in refrigerator at 6 ± 2 °C for six months. After 6 months, each sample was examined for visual appearance and fully evaluated as previously done for the corresponding fresh one. Results

of the stored samples were compared to those of the corresponding fresh ones. Student's t-test was applied using SPSS 17.0® software (2008, IBM SPSS Statistics, NY, USA) to relate mean values where differences were considered significant at $p < 0.05$. To compare the release profiles of the drug from the stored samples to those from the corresponding fresh ones, the similarity factor (f_2) was determined following the next equation **[1]**:

$$f_2 = 50 \times \log \left[\left\{ 1 + \left(\frac{1}{n} \right) \sum_{t=1}^{n} (R_t - T_t)^2 \right\}^{-0.5} \times 100 \right]$$

Where n is the number of sampling points, R_t and T_t are the mean % of drug released at time t from fresh and stored samples; respectively.

Results and Discussion

After six months-storage at 6 ± 2 °C, both the optimal zolmitriptan-loaded bilosomes and the prepared mucoadhesive in-situ gelling system containing it didn't show any change in visual appearance (color and texture). For all the assessed parameters, the mean values calculated for the stored samples were statistically non-significantly ($p > 0.05$) different from those for the respective fresh ones (data not shown).

By comparing the in-vitro release profiles of the stored samples and the corresponding fresh ones, it was apparent that both profiles were almost super-imposed. This was proven by the calculated values of similarity factor (f_2) which were calculated to be 86.20 for the optimal bilosomes and 88.04 for the mucoadhesive in-situ gelling system. As all calculated values were greater than 50; it could be concluded that storage of the investigated samples did not influence the release behavior of the drug [1].

References

1. Moore, J.W.; Flanner, H.H. Mathematical comparison of dissolution profiles. *Pharm. Tech.* **1996**, *20*, 64-74.
